# An exploratory study on the preparation of metallic rhenium (Re) and ReO_3_ via non-contact solution plasma electrolysis

**DOI:** 10.1371/journal.pone.0338178

**Published:** 2025-12-08

**Authors:** Hong Fu, Lingling Shen, Zhongning Shi, Nan Zhou, Qiangqiang Cheng, Wanan Lai

**Affiliations:** 1 School of Intelligent Manufacturing, Jiangsu Vocational Institute of Architectural Technology, Xuzhou, China; 2 School of Mines, China University of Mining and Technology, Xuzhou, China; 3 School of Metallurgy, Northeastern University, Shenyang, China; 4 School of Architectural Construction, Jiangsu Vocational Institute of Architectural Technology, Xuzhou, China; University of the Witwatersrand Johannesburg, SOUTH AFRICA

## Abstract

Metallic rhenium (Re) and its oxides (e.g., rhenium trioxide ReO_3_) have been extensively utilized in fields including national defense, aerospace, and electronics, owing to their unique “Re effect” and favorable thermal expansion characteristics. However, conventional preparation methods confront substantial scientific and technical challenges, primarily arising from the intrinsic physicochemical properties of Re and the complexity of synthesis processes. This study focused on the synthesis of ultrafine Re/ReO_3_ composite particles via plasma electrolysis in aqueous solutions, employing a Re-containing precursor as the anode feedstock and a tungsten (W) wire as the cathode. The effects of key process parameters, including discharge voltage, electrolyte composition, solution acidity, and electrolysis duration on the morphological features of the resultant particles were systematically investigated. Additionally, plasma emission spectra, discharge behavior (encompassing cathode/anode voltage variations), electrolyte volume, and temperature evolution were monitored to elucidate the reaction mechanisms and dynamic behaviors governing the synthesis of different ultrafine powders. The results demonstrated that ReO_3_^-^ Re composite powders can be successfully prepared using this method. Notably, reducing the discharge voltage suppressed W sputtering from the cathode and promoted the formation of ReO_3_ as the dominant phase. Furthermore, the addition of H_2_SO_4_ mitigated particle aggregation by enhancing hydrogen-bonding coordination among particles and electrolyte components.

## Introduction

Rhenium (Re), a critical refractory metal renowned for its exceptional thermal stability and superior mechanical properties, has become an indispensable material in advanced technological applications. Discovered in 1925, Re’s scarcity (0.4 mg/kg in Earth’s crust) and strategic importance in industries such as aerospace, electronics, and nuclear energy have fueled global demand [1–3]. Additionally, Re-Ni and Mo-Re alloy can stand a repeated heating and cooling process without loss of mechanical property, which make Re and its compound wildly used in the field of national defense, aerospace and electronics [4–7]. Another notable property of Re is its ability to enhance the ductility of W-Re and Mo-Re alloys while lowering their ductile-brittle transition temperature—a phenomenon commonly referred to as the “Re effect” [8–11]. Additionally, ReO_3_ is considered as one of the most special transition metal oxides since it has a low electrical resistivity, which can be comparable to copper and silver [12]. And it can exhibit negative thermal expansion (NTE) behavior and metallic conductivity—has garnered attention for catalytic and optoelectronic applications [13–17].

Despite its potential, the synthesis of metallic Re and ReO_3_ faces significant challenges. In general, there are three principles to produce metal Re, one is pulsed-laser decomposition [14], hydrogen reduction of ammonium perrhenate (NH_4_ReO_4_) [18], and another method is electrochemical deposition [19]. Among these, pulsed-laser decomposition has received limited attention due to its high energy consumption and poor scalability. In contrast, H_2_ reduction and electrochemical deposition have been the focus of extensive research. The H_2_ reduction process involves the gradual deoxidation of NH_4_ReO_4_ following its thermal decomposition, requiring precise control of temperature and O_2_ partial pressure to ensure pure Re formation. However, this method typically yields Re powders with irregular morphologies and suboptimal sinter ability. While, electrochemical deposition provides a much milder approach. Research in KF-KBF_4_-B_2_O_3_ at 773 K is conducted, and the result showed that the process of rhenium cathode reduction can be described as a one-step reaction Re(VII) → Re, and rhenium electrodeposition presumably occurred from two types of complex rhenium ions (KReO_4_ and K_3_ReO_5_). Electrochemical deposition is identified as an effective way to produce Re particles, it faces a critical limitation: the low overpotential for hydrogen evolution in aqueous solutions leads to H_2_ adsorption on the cathode surface, hindering the formation of high-quality Re films [20]. Besides, the pH value and the potential must be controlled precisely for the preparation process. Several researches have focused on the production of Re and its alloy including in acid and alkaline solution.

The challenge currently is that the production for Re and its oxides needs gas carrier and professional equipment such as hydrogen and tube furnace for the process of H_2_ reduction. Thus, there is an urgent need to develop a low-cost, facile synthesis strategy with simplified experimental conditions.

Recent advances in non-equilibrium plasma technologies have opened new avenues for advanced material synthesis. Solution plasma electrolysis (SPE) represents an unconventional electrochemical technique that differs from traditional electrolysis by generating a mixture of high-energy species at the cathode [21–23]. Specifically, the cathode in SPE enables localized generation of high-energy electrons under mild conditions (ambient temperature and pressure), facilitating the formation of metastable phases and nanostructured materials that are difficult to obtain via conventional methods. Unlike thermal plasma (which operates at temperatures >1000°C) and glow discharge (which requires low-pressure gas environments), SPE integrates traditional electrolysis with plasma physics to drive chemical reactions under ambient conditions, significantly reducing energy consumption and equipment complexity [24,25]. A comparative summary of SPE and other plasma-based techniques is provided in Table 1.

**Table 1:** Difference between solution plasma and thermal plasma, glow discharge.

Technique	Operating Conditions	Applications
**Solution Plasma SPE**	Ambient temperature/pressure	Metal/oxide synthesis, nanomaterials
**Thermal Plasma**	High temperature (>1000°C)	Bulk material processing
**Glow Discharge**	Low-pressure gas environment	Thin-film deposition

Cathodic plasma discharge in aqueous solutions has emerged as a novel approach for fine powder synthesis. Its key advantages include: (1) high cathode voltages that accelerate electrons to generate reactive species, promoting critical reaction steps; (2) low discharge currents (typically in the milliampere range), ensuring low energy consumption; and (3) excellent controllability over particle morphology and size via parameter tuning [26–29]. To date, SPE has been successfully applied to synthesize metals (Cu, Ni, Pt, Al), alloys (Sm-Co, Co-Pt), and metal oxides (WO_3_, ZrO_2_) [26–29]. However, systematic studies on Re-based material synthesis via SPE remain scarce, especially compared to the mature exploration of oxides/sulfides in the following works [30–35]. For example, the synthesis of functional metal-based materials (oxides, sulfides, composites) has been extensively explored via other mild strategies, with metal-organic frameworks (MOFs) and their derivatives standing out for tunable porous structures and multi-component synergy, which motivates our exploration of SPE for Re/ReO_3_ synthesis to fill this gap. To our knowledge, this represents one of the earliest systematic studies on rhenium-based particle synthesis using solution plasma electrolysis. Previous plasma-in-liquid works have demonstrated the synthesis of several types of metals and various oxides, but rhenium remains scarcely explored. Our study extends this methodology to Re/ReO_3_ and highlights its feasibility. This research aims to investigate the roles of H-bonding interaction in suppressing particle aggregation, and demonstrate the impact of sulfate additives on discharge efficiency and product dispersion.

Although plasma electrolysis for ReO_3_ synthesis is relatively unexplored, our work extends the plasma electrochemical synthesis method to ReO_3_, highlighting its potential for controlled phase and morphology. This comparison with other metal oxide syntheses emphasizes the uniqueness of our approach and provides a solid foundation for future work on ReO_3_ and other refractory metal oxides. Our work addresses critical gaps in Re synthesis by integrating plasma physics, electrochemistry, and materials science. The outcomes hold promise for advancing Re-based technologies in high-temperature alloys, catalysts, and energy storage systems.

## Experimental

### Materials and methods

All of the chemical reagents used in the experiments were analytical grade and were used without any further purification. In this study, the Re origin was NH_4_ReO_4_ aqueous solution. The electrolytic cell configuration consisted of a 1 mm-diameter tungsten (W) wire cathode and a helical platinum (Pt) wire anode (dimensions: 1 × 15 × 25 mm). In the typical experimental process, the investigated solution of 0.05M and 0.2M NH_4_ReO_4_ were prepared first. After that, polishing electrode and assembling the experimental equipment were done. The last procedure comes to set up the discharge voltage. With the increase of voltage, the electrolysis changes from normal electrolysis to plasma discharge accompanied with emission of light and heat. To assess the effect of electrolyte composition, additional experiments were conducted with electrolytes containing H_2_SO_4_ or NaCl as additives, using the same cell configuration [26].

### Product collection and characterization

After discharge electrolysis, the powder was collected with centrifuge (300 rpm/min), and then rinsed with distilled water three times and dried in vacuum drying oven and vacuum freezing drying oven respectively to explore the effect of dry procedure on the product composition and morphology. The X-ray diffraction (XRD, X’Pert Pro using Cu Ka radiation) was used to identify the phase of product, and scanning electron microscopy/ energy dispersive X-ray spectroscopy (SEM/EDS, Ultra Plus) was employed to observe the morphology and element distribution. Emission spectrum instrument (Ocean Maya 2000 Pro) was to detect the excited species generated in plasma discharge process. The specific details about above mentioned instruments can be found in previous study [26–28]. A Thermofisher Evolution 220 was used to record the UV-vis spectra of the prepared product.

## Results and discussion

### Experimental results of Re and ReO_3_

**Fig 1:**
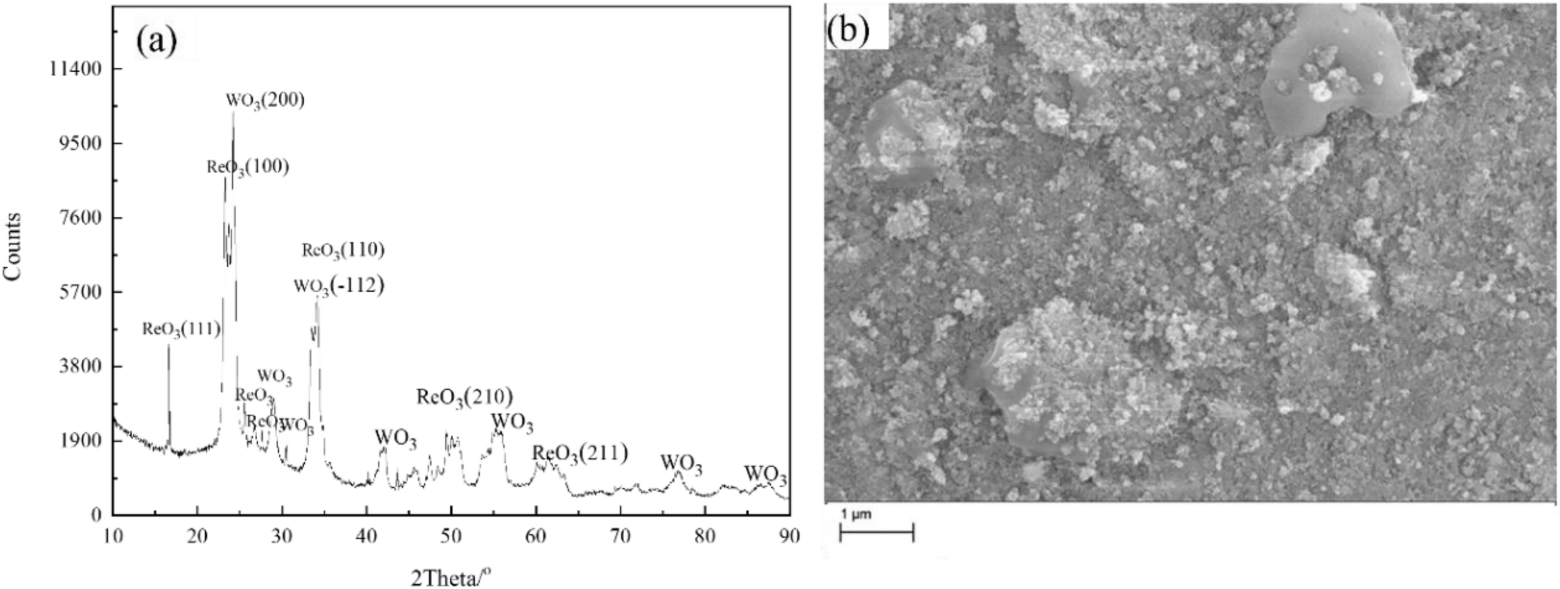
XRD pattern and SEM image obtained in solution of 0.05M NH_4_ReO_4_ at 320V, W wire locates 6 mm above the surface of the electrolyte at room temperature.

Initial experiments were conducted in 0.05 M NH_4_ReO_4_ solution at an applied voltage of 320 V. The generated powder was analysed by XRD as shown in Fig 1(a). From the XRD pattern, it can be found that the product is composed of ReO_3_ and WO_3_. The diffraction peaks of WO_3_ and ReO_3_ are almost in the same intensity. The SEM image in Fig 1(b) shows that the product has two types of morphology. One is particle shape with diameter of 30nm, and another morphology of bulk shape is found.

**Fig 2:**
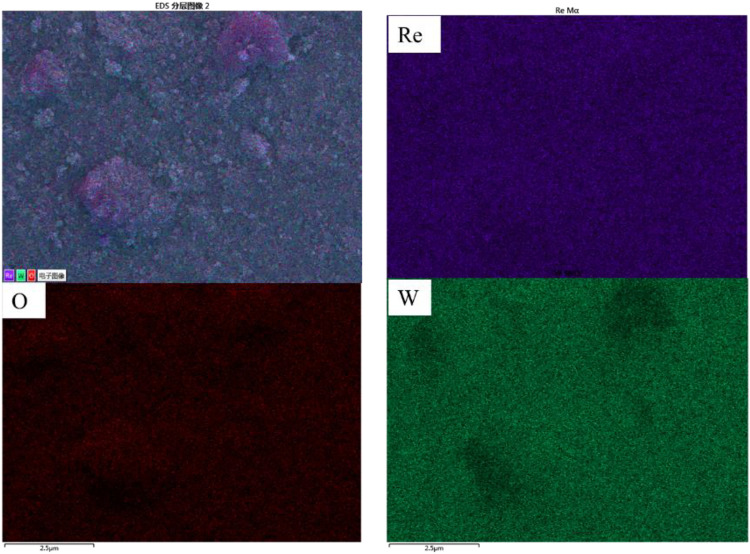
Element distribution of the corresponded section in Fig 1.

To clarify the composition of the two morphological fractions, EDS elemental mapping was performed on the selected region (Fig 2). The results showed a clear segregation of Re and W: W was absent in the bulk aggregates, whereas Re and O were concentrated on the surface of these aggregates. Besides, element Re was distributed uniformly in the entire selected section. Moreover, EDS analysis based on two types of morphology is conducted. The result shows that the atomic ratio of Re, W and O is 40:12:48 and 16:13:71, respectively for bulk and particles shape. Combined with the XRD pattern, it can be concluded that metal Re maybe generated in the discharge electrolysis process except for ReO_3_ and WO_3_. The reason for generation of WO_3_ is contributed to thermal sputtering of cathode.

The mechanism of W sputtering is as follows: during plasma discharge, the W cathode generates high-energy electrons via field emission, which ionize the electrolyte and produce reactive species (e.g., H^+^, ReO_4_^-^, and free radicals) [30]. And during this process, high-energy electrons and Joule heating elevate surface temperatures, overcoming W’s surface binding energy and driving atomic/molecular ejection, which leads to thermal sputtering of W. High-energy electrons and Joule heating elevate surface temperatures, overcoming W’s surface binding energy and driving atomic/molecular ejection [36,37]. The thermal sputtered W electrode enters into electrolyte solution and collected. The result matches well with SEM images in Fig 1a, and EDS analysis in Fig 2. In addition, during plasma discharge, high-energy electrons bombard the electrolyte surface, ejecting droplets that interact with the W electrode. These droplets facilitate the adsorption of ReO_4_^-^ ions onto W surfaces, enabling controlled nucleation of Re-based particles [38]—a phenomenon also observed in our previous work on other metal oxide synthesis [27]. It should be pointed out that the role of working electrode as a substrate should be closely related to electrolysis parameters and electrolytes. In this study, the role of the electrolytic substrate was not particularly prominent.

**Fig 3:**
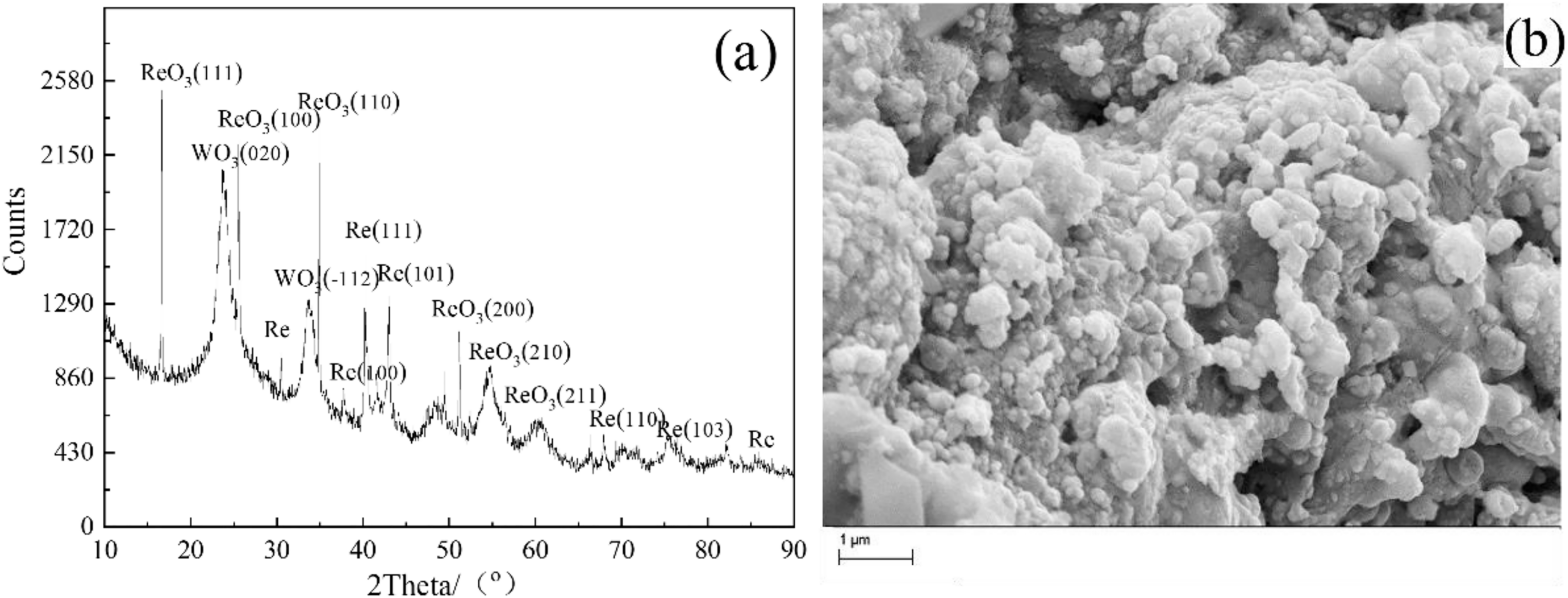
(a) XRD pattern and (b) SEM image obtained in solution of 0.1M NH_4_ReO_4_ at 280V for 30 min, W wire locates 6 mm above the surface of the electrolyte at room temperature.

During discharge in 0.05 M NH_4_ReO_4_, the segment of the W wire located within 6 mm of the discharge tip became noticeably heated, despite a low discharge current (~0.1 A). The reason probably derived from the low conductivity of the electrolyte. To address this, subsequent experiments were conducted in 0.1 M NH_4_ReO_4_ at a reduced voltage of 280 V. As shown in Fig 3a, increasing the NH_4_ReO_4_ concentration from 0.05 M to 0.1 M altered the phase composition: The phase of the product undergoes a change, with the phase dominated by the WO_3_ diffraction peak transforming into the phase dominated by ReO_3_, and a diffraction peak of metal Re appearing in the product. Under these conditions, the SEM image of the product shows spherical particles clustered together, and there are also small particles on the surface that appear to be wrapped by electrolyte or water molecules. Compared to the study of low concentration electrolytes, the particle size is larger, ranging from 300 to 500 nm. The composition of the particles was analyzed by EDS, and the results showed that the atomic ratio of Re: W: O was 24:10:66. Compared with the results of electrolysis at low concentrations, the proportion of W in the product of 0.1M electrolyte has decreased, and the atomic ratio of Re to O is greater than 1:3, indicating the presence of metal Re in the product, which is consistent with the XRD characterization results.

**Fig 4:**
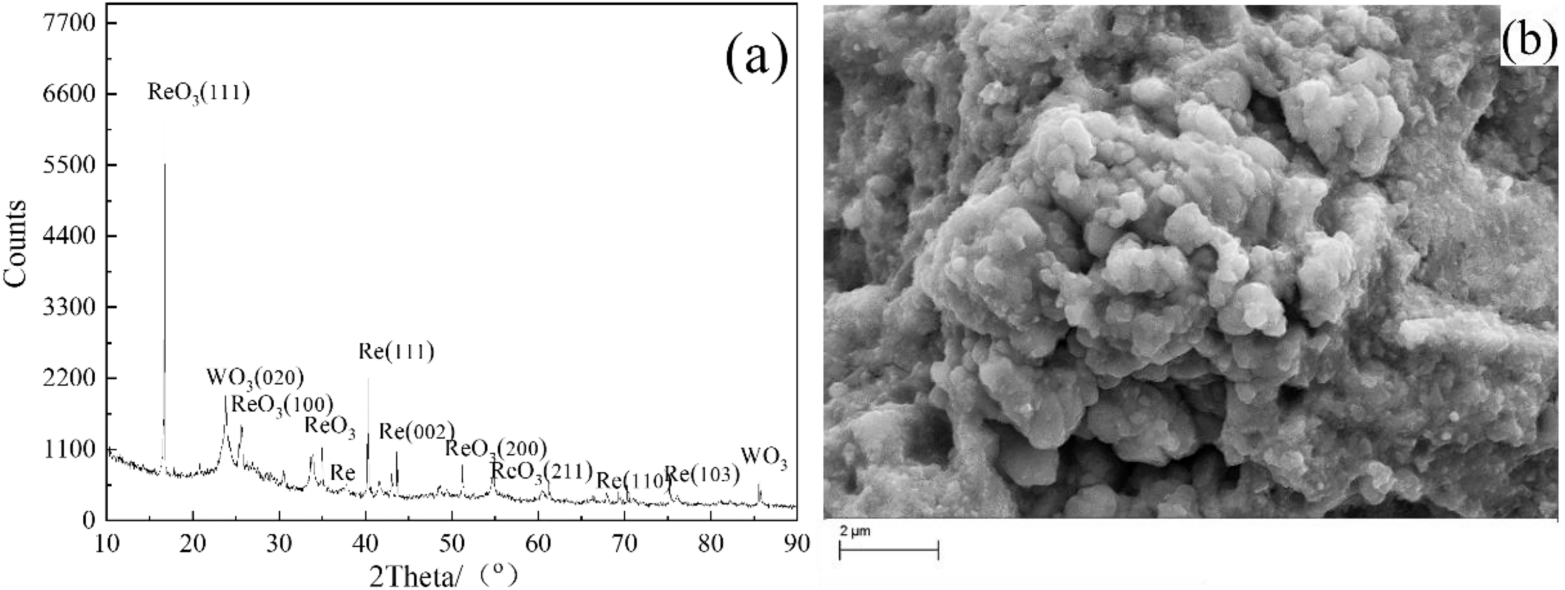
(a) XRD pattern and (b) SEM image obtained in solution of 0.2M NH_4_ReO_4_ at 230V for 30 min, W wire locates 6 mm above the surface of the electrolyte at room temperature.

When the experiment was conducted in 0.2M NH_4_ReO_4_, and the applied voltage decreases to 230V. The XRD patter shows an obvious difference in terms of the phase composition in the product in Fig 4. It can be found that the product is composed of ReO_3_, Re and WO_3_. Compared with the pattern in Fig 1, it can be found that the strongest diffraction peak changes more obviously from WO_3_ to ReO_3_ phase. The diffraction peaks of WO_3_ fades extremely, moreover, it can be found the characteristic peaks of metal Re stronger. From the magnified image shown in Fig 4(b), it can be found that the obtained product was sphere like but with strong aggregation. EDS analysis result indicates that the atomic radio of Re, W and O is 30:9:61, which means that the W content in synthesized powder decrease when the discharge was conducted in 0.2M NH_4_ReO_4_. Under the best low-W condition of 0.2 M NH_4_ ReO_4_, 230 V, rietveld-type fitting of the XRD data revealed that the phase fractions were approximately 60–65% ReO_3_, 20–25% Re, and less than 15% WO₃. These results support the dominance of ReO_3_ at lower voltages. Future work involving TEM/SAED would further validate these phase fractions at the nanoscale. During the discharge process in 0.2M NH_4_ReO_4_, the cathode shows no obvious hot phenomenon, and the current stables around 0.1A. The size of the electrolytic product is more uniform compared with that obtained in 0.1M NH_4_ReO_4_, without the presence of small particles, with particle sizes ranging from 300 to 400nm.

It is found that increasing the concentration of electrolyte can decrease the discharge voltage, however the synthesized particles aggregate seriously. W content in the products decreased significantly with the decrease in voltage. This trend was observed in our SEM/EDS analysis of the products obtained at varying voltages (Figs 2–4). At higher voltages of 320 V, the product showed more WO_3_, which can be attributed to thermal sputtering of the W cathode. In contrast, at lower voltages of 230 V, the proportion of WO_3_ in the product was reduced, and the dominant phase became ReO_3_, as confirmed by XRD and EDS analysis. Measured mean dimeter extracted from SEM images in Figs 1, 3 and 4 as shown in S1 Fig. The mean diameter can be calculated as 124nm ± 45nm, 236nm ± 186nm, 217nm ± 125nm. It should be noticed the obtained data showed a big standard deviation due to coincidentally existing and a small amount of big particle. The SEM images revealed that the morphology of the product was consistent with the sputtering of W from the cathode. At high voltages, we observed large, irregularly shaped WO_3_ particles, while at lower voltages, spherical ReO_3_ particles were the predominant product.

**Fig 5:**
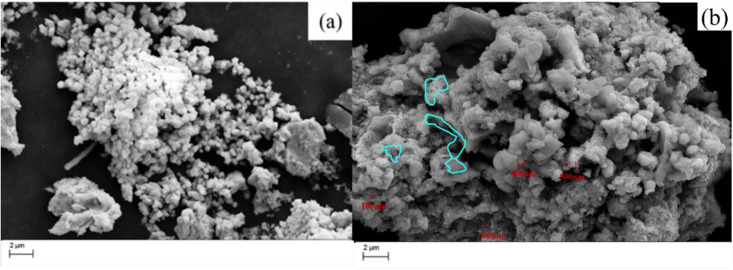
SEM images of powder obtained in solutions (a) 0.2M NH_4_ReO_4_ – 0.2M H_2_SO_4_, (b) 0.2M NH_4_ReO_4_ – 0.6M H_2_SO_4_, W wire locates 6 mm above the surface of the electrolyte at room temperature.

To mitigate particle aggregation, experiments were conducted with H_2_SO_4_ as an additive (building on our previous findings for other metal oxides [26,27]). Two electrolyte systems were tested: 0.2 M NH_4_ReO_4_ - 0.2 M H_2_SO_4_ and 0.2 M NH_4_ ReO_4_ −0.6 M H_2_SO_4_. The SEM images (Fig 5a and 5b) showed spherical particles with reduced aggregation and a size range of 200–700 nm. When the H_2_SO_4_ concentration was increased to 0.6 M, the product exhibited a porous, loose structure with fine particles (~100 nm) uniformly distributed (Fig 5b). This observation confirms that H_2_SO_4_ plays a critical role in suppressing. It can be found many fine particles in a diameter around 100nm. From the result of discharge in solution contained H_2_SO_4_, it can be concluded that the addition of H_2_SO_4_ plays an important role during the discharge process exactly as our research before. The product is synthesized in a different formation mechanism and exhibits a distinguished morphology due to the discharge of H^+^ in H_2_SO_4_. The reduced aggregation in H_2_SO_4_ solutions cannot be attributed to pH alone. Control experiments in NaCl solutions of comparable ionic strength did not suppress aggregation, suggesting that sulfate ions, rather than merely acidity, play a crucial role in stabilizing particle dispersions. This interpretation aligns with prior findings that sulfate ions stabilize colloids through hydrogen-bonding mechanisms. Besides, it should be noticed that this observation has been mentioned in our previous report [28]. It may attribute to the H-bond function by coordinating between the aggregations, which has been demonstrated in the research of solvents extract [39,40]. Although the phenomenon in our research is not exactly the same as that happened in other research, actually we believe that the H-bond coordination happens not in the discharge process but the prepared particles aggregation in our research just like the research mentioned above.

EDS analysis shows that the atomic radio of Re/W/ O is 47:5:48, and it can be inferred from this that the mole radio of Re/ReO_3_ is 11:36. It can be found that the intensity of diffraction peaks of ReO_3_ and WO_3_ decrease from the XRD pattern, but that of Re keep almost the same as that of obtained in solution contained 0.2M H_2_SO_4_, which goes against the result of EDS analysis. It is probably caused by the poor crystallinity of Re, and it shows weak intensity in XRD pattern.

### Formation mechanism of Re and ReO_3_

Considering the discharge process, three possible ways can be responsible for formation of ReO_3_. One derives from oxidation of generated Re, and the other results from discharge electrolysis of NH_4_ReO_4_. Besides, ReO_3_ should be produced from contamination of condensed production located on top of cap and corundum tube, because some powder can be found from the cap and tube after discharge.

**Fig 6:**
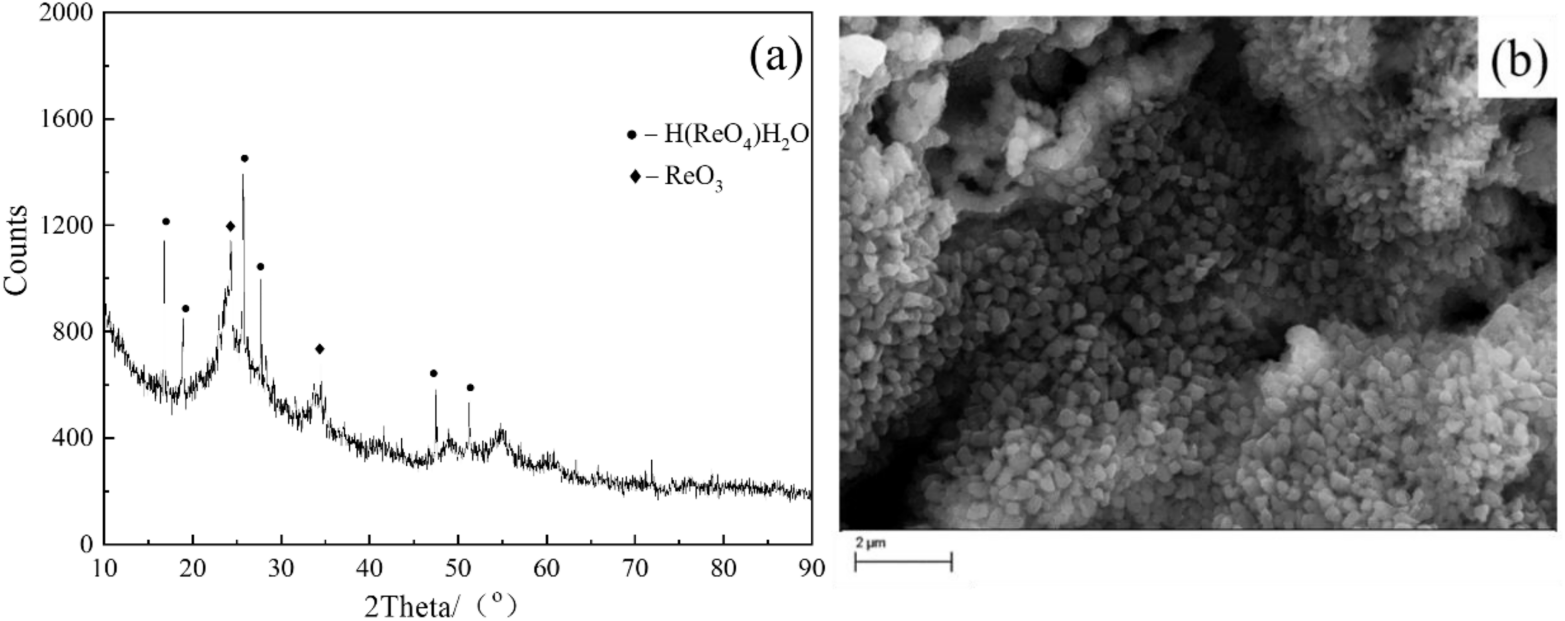
XRD pattern and (b) SEM image obtained in 0.2M NH_4_ReO_4_ – 0.2M H_2_SO_4_ with anode and cathode shielded, W wire locates 6 mm above the surface of the electrolyte at room temperature.

Considering the possibility of oxidation by oxygen and vapour. An experiment that both of anode and cathode are shielded by a quartz tube was conducted in solution 0.2M NH_4_ReO_4_−0.2M H_2_SO_4_. After the experiment, the powder was collected and characterized by XRD as shown in Fig 6. The collected powder was charactered by XRD and SEM as shown in Fig 6. It can be found that there are two phases in the condensed product, ReO_3_ and H (ReO_4_) H_2_O in Fig 6(a). Among them, ReO_3_ comes from reduction of ReO_4_^-^, and the formation of H (ReO_4_) H_2_O is due to the combination of sputtered electrolyte with hydrogen ions and water molecules under the action of thermal field. The morphology of the product is polyhedral particles with uniform size, with a particle size of around 400 nm as shown in Fig 6(b). In addition, the XRD result shows that ReO_3_ still exists in the generated product, and the peaks of ReO_3_ keep its intensity compared with that obtained in equipment without the tube. In order to make sure the reaction tendency between as-prepared Re and O_2_ as well as H_2_O.

**Fig 7:**
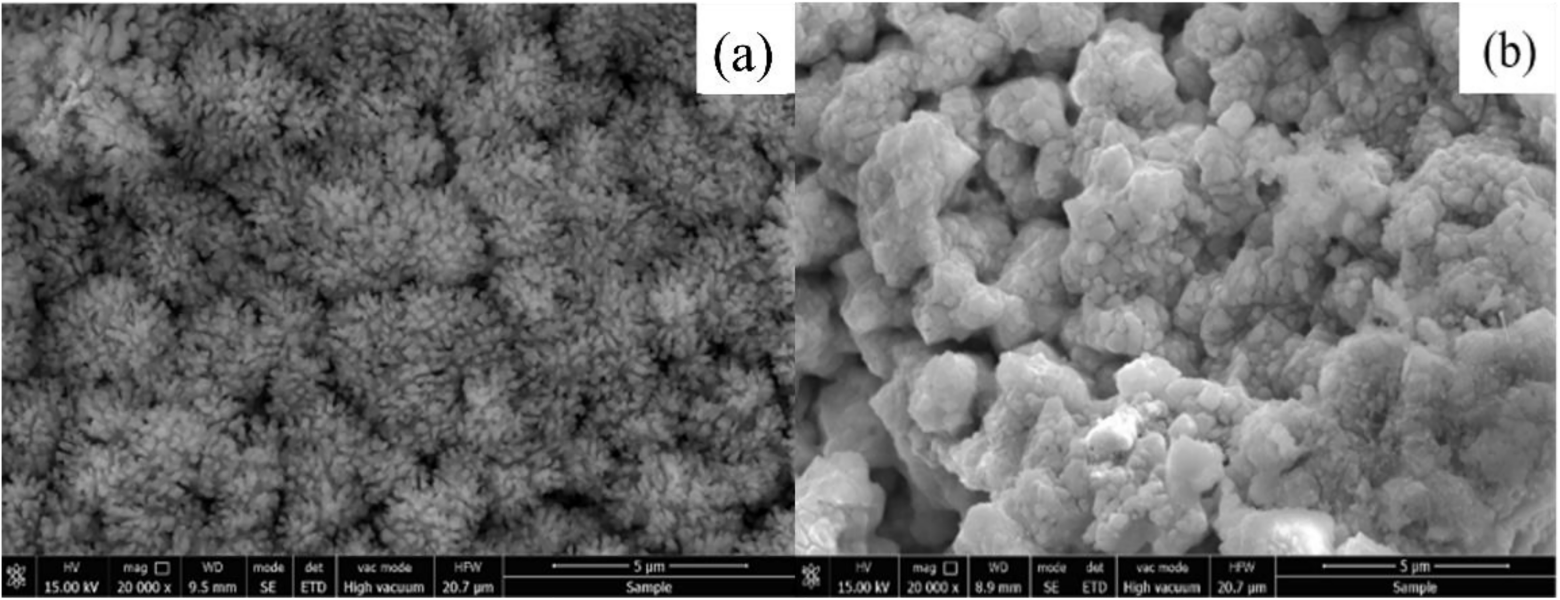
SEM images of powder obtained in solutions with freeze dryer (a) 0.1M NH_4_ReO_4_ – 0.2M H_2_SO_4_, (b) 0.2M NH_4_ReO_4_ – 0.2M H_2_SO_4._

Adding sulfuric acid can reduce particle aggregation, but during the drying process of the collected product, a film will form on the surface of the particles due to their activity. Therefore, freeze-drying is used to treat the centrifuged product. Firstly, the wet suspension after centrifugation is frozen, and the product is dried at low temperature and under certain vacuum conditions using the sublimation process of water in the freezer. During this process, water molecules sublime directly (by passing the liquid phase), which enables the electrolyzed particles to retain their original morphological features rather than maintaining an “electrolytic state”. The plasma discharge was conducted in solution of 0.1M NH_4_ReO_4_ − 0.2M H_2_SO_4_ and 0.2M NH_4_ReO_4_ − 0.2M H_2_SO_4_, after collection and dry the morphology was shown in Fig 7. As shown in Fig 7(a), the product exhibits a flower-like morphology composed of rod-shaped subunits, with each rod measuring approximately 500 nm in length and 100 nm in diameter. Furthermore, the product in Fig 7(b) displays distinct interparticle boundaries without significant aggregation, confirming that both collection and drying processes exert a critical influence on particle morphology. SEM comparisons of oven-dried and freeze-dried samples (Fig 7) indicate that freeze-drying preserves particle separation and prevents film formation, while oven-dried samples show aggregation due to surface activity. This demonstrates that drying methods play a crucial role in determining the final morphology of the product, highlighting the importance of post-processing conditions in particle synthesis. However, the reason needs to be investigated in detail in our further study.

**Fig 8:**
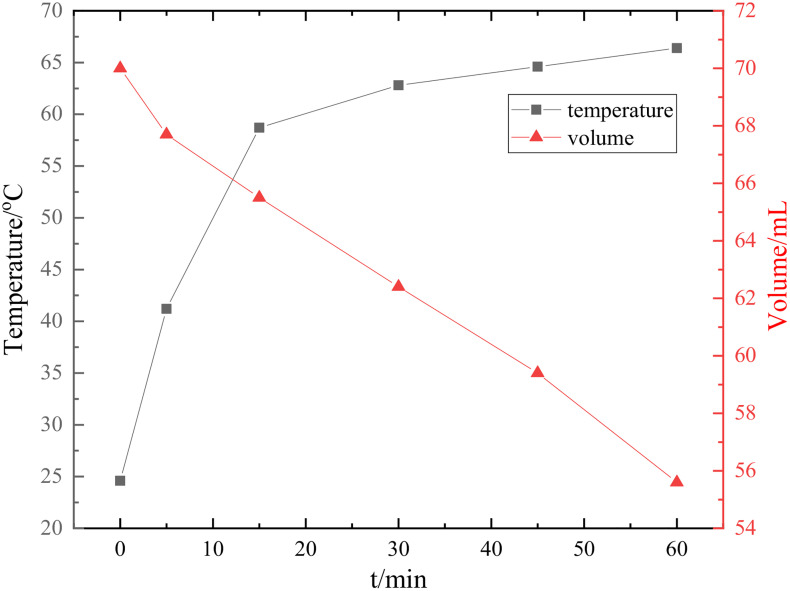
Relationship between temperature and volume of electrolyte measured in the plasma discharge process.

To elucidate the formation mechanisms of Re and ReO₃, the discharge process was systematically investigated by analyzing the relationships between electrolyte temperature, cathode voltage, plasma emission spectra, and UV-vis absorption spectra. First, the relationships between temperature as well as volume of electrolyte and time are shown in Fig 8. From the curves it can be found that the temperature raises with discharge time from 24.6 °C to 68.4 °C, especially an extremely increase during the start 15 min. But the increase rate slows down after15 min. The volume of bath shows an opposite tendency in the whole discharge period between 70 mL and 54.6 mL. The volume changes slightly at first, while it vapors a lot after 30 min. This phenomenon is attributed to the extreme temperature rise in the region directly beneath the plasma discharge zone, which enhances the evaporation rate of the electrolyte. During the initial discharge stage, a large temperature gradient exists between the plasma-adjacent region and the bulk electrolyte, facilitating efficient heat conduction and leading to a rapid increase in bulk temperature. Once the bulk electrolyte reaches a certain temperature, the temperature gradient narrows, weakening heat conduction and slowing the rate of temperature rise. With the discharge proceeding, the energy puts into the solution continuously, which leads to the vapor of the area just below plasma area accelerates. Therefore, the loss of bath volume increases.

**Fig 9:**
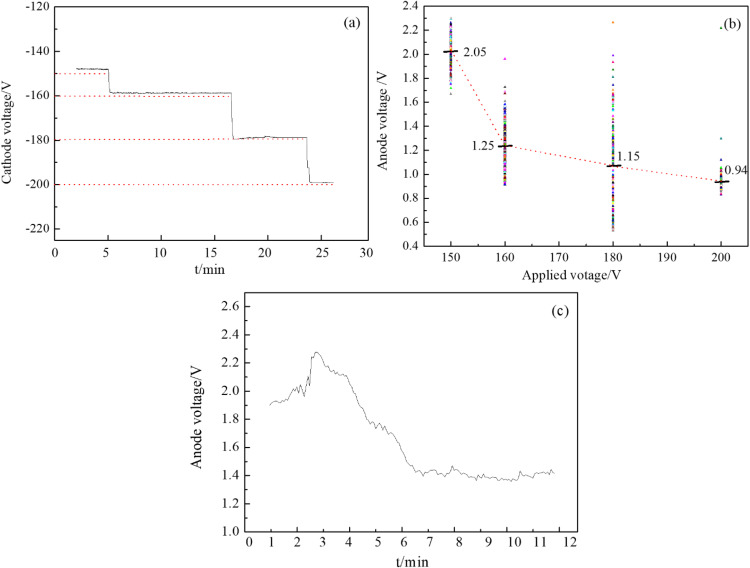
Measurement of (a) cathode and (b) (c) anode voltage in plasma discharge electrolysis.

During the discharge process, a voltage of 150-200V was applied, with Pt as the reference electrode. The cathodic and anodic voltage curves were measured over time, and the results are shown in Fig 9.

Cathode voltage values were measured by applying different voltages of 150 V, 160 V, 180 V and 200 V. As shown in Fig 9(a), when a voltage of 150 V is applied, the average cathode voltage is 147.95 V; when the voltage is increased to 160 V, the average cathode voltage rises to 158.75 V; and at 200 V, the cathode voltage reaches 199.06 V. By subtracting the measured cathode voltage from the applied voltage, the anode voltage was derived, as shown in Fig 9(b). It is evident that the anode voltage varies with the applied electrolysis voltage, decreasing as the applied voltage increases: specifically, the anode voltage was 2.05 V at 150 V, decreased to 1.25 V at 160 V, and further dropped to 0.94 V at 200 V. Direct measurements of the anode voltage are presented in Fig 9(c). Initially, the anode voltage shows an upward trend during electrolysis, gradually stabilizing over time with an average value of 1.43 V. Regardless of the applied voltage, the anode voltage remains between 1-2V, with the voltage drop primarily occurring in the cathode plasma region. Kaneko’s research on potential distribution during plasma electrolysis reveals that when plasma functions as the cathode, a notable cathode pressure drop occurs [41]. In contrast, no significant anode voltage drop is observed when plasma acts as the anode. This finding is consistent with our results, suggesting that plasma at the cathode emits a large number of high-energy particles toward the liquid surface, whereas plasma at the anode only generates a small number of low-energy electron clusters on the liquid surface.

**Fig 10:**
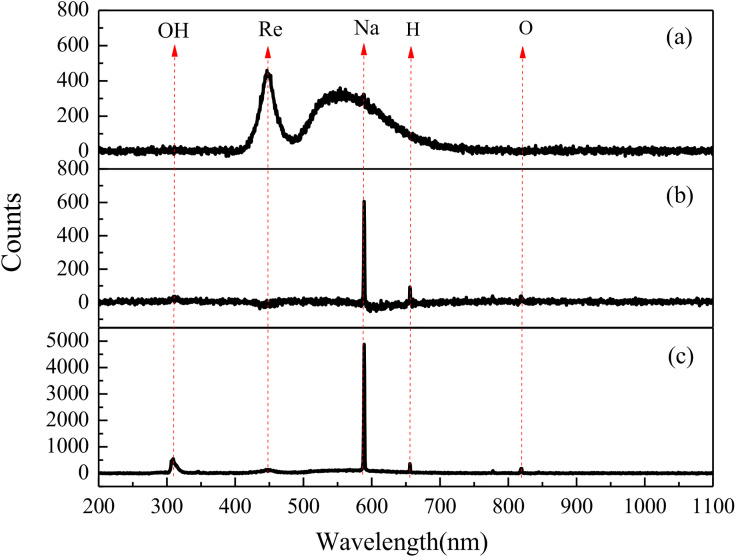
Emission spectrum obtained in 0.1M NH_4_ReO_4_ – 0.2M H_2_SO_4_ solution.

Plasma emission spectra were collected at 1-second intervals during discharge and can be categorized into the three types described above, as shown in Fig 10. The data reveals that the emission peaks observed at 588 nm were attributed to the discharge of Na atoms, while signals at 327 nm and 656 nm correspond to OH radicals and H atoms, respectively. The spectral features were identified based on established line databases. the addition of NaCl to the solution leads to the generation of Na atoms during cathodic discharge. Throughout the electrolysis process, both Na and H ions undergo discharge. However, in Fig 10(a), the broadening of the Na transition peak covers the signal from H element transitions, making the emission spectrum peak of H atoms less prominent. In addition, signals of OH radicals can also be observed, but their intensity is weak, indicating that OH discharge is not a priority ionization process. The emission spectrum data presented in Fig 10 further illustrates that the probability of Re element transition during plasma discharge electrolysis is very low, which is also an important reason for the low presence of metallic Re in prepared powder. Because the source of rhenium in electrolyte is ReO_4_^-^, and the negative ion groups will move towards the anode under electric field. Therefore, it is difficult to discharge ReO_4_^-^ ions directly at the cathode. Future work could include optical-emission actinometry to provide a more quantitative comparison of emission intensities and to better correlate these measurements with the yield and energy efficiency of the process.

However, due to the presence of H^+^ in the solution, reactions may occur by equations (1)-(3).


$${{\text{ReO}}_{\text{4}}}^{-}+{\text{2H}}^{+}⇌{{{\text{H}}_{\text{2}}\text{OReO}}_{\text{3}}}^{+}\;(\text{fast})$$ (1)

$${{{\text{H}}_{\text{2}}\text{OReO}}_{\text{3}}}^{+}⇌{\text{H}}_{\text{2}}\text{O}+{{\text{ReO}}_{\text{3}}}^{+}\;(\text{slow})$$ (2)

$${{{\text{H}}_{\text{2}}\text{OReO}}_{\text{3}}}^{+}+\text{n}{\text{H}}_{\text{2}}\text{O}⇌{\left({\text{H}}_{\text{2}}\text{O}\right)}_{\text{n}+\text{1}}\;{{\text{ReO}}_{\text{3}}}^{+}\;(\text{slow})$$ (3)

From the above reactions, it can be inferred that ReO_4_^-^ rapidly combines with H^+^ in acidic solution to undergo a reversible reaction (1), forming H_2_OReO_3_^+^. Due to the slow hydrolysis rate of H_2_OReO_3_^+^ to ReO_3_^+^ (Eq. (2)), the primary Re-containing species in the solution are ReO_4_^-^, H_2_OReO_3_^+^, and ReO_3_^+^. Among these, H_2_OReO_3_^+^ and ReO_3_^+^ are the main species that migrate to the cathode discharge zone for subsequent reduction, as ReO_4_^-^ is repelled by the cathode. However, compared to Na^+^ and H^+^, these two groups have larger volumes, which greatly reduces their discharge frequency and consequently leads to fewer ionization cycles of rhenium element. As shown in Fig 10, the emission spectrum of rhenium element is very weak. H_2_OReO_3_^+^and ReO_3_^+^can be further reduced by electrons, resulting in reactions (4) and (5) to generate ReO_3_. ReO_4_^-^ can also be directly reduced under acidic conditions, and the main reactions that occur in this process are as follows equations (6) to (9):


$${{\text{ReO}}_{\text{3}}}^{+}+{\text{e}}^{-}={\text{ReO}}_{\text{3}}$$ (4)

$${{{\text{H}}_{\text{2}}\text{OReO}}_{\text{3}}}^{+}+{\text{e}}^{-}={\text{ReO}}_{\text{3}}+{\text{H}}_{\text{2}}\text{O}$$ (5)

$${{\text{ReO}}_{\text{4}}}^{-}+{\text{2}\text{H}}^{+}+{\text{e}}^{-}={\text{ReO}}_{\text{3}}+{\text{H}}_{\text{2}}\text{O}$$ (6)

$${{\text{ReO}}_{\text{4}}}^{-}+{\text{8}\text{H}}^{+}+\text{7}{\text{e}}^{-}=\text{Re}+\text{4}{\text{H}}_{\text{2}}\text{O}$$ (7)

$$\text{2}{{\text{ReO}}_{\text{4}}}^{-}+{\text{1}\text{0}\text{H}}^{+}+\text{8}{\text{e}}^{-}={{\text{Re}}_{\text{2}}\text{O}}_{\text{3}}+\text{5}{\text{H}}_{\text{2}}\text{O}$$ (8)

$${{\text{ReO}}_{\text{4}}}^{-}+{\text{4}\text{H}}^{+}+\text{3}{\text{e}}^{-}={\text{ReO}}_{\text{2}}+{\text{2H}}_{\text{2}}\text{O}$$ (9)

$${{\text{ReO}}_{\text{4}}}^{-}+{\text{e}}^{*-}=\text{Re}+\text{2O}+{\text{O}}^{\text{2}-}$$ (10)

$${{\text{ReO}}_{\text{4}}}^{-}+{\text{3e}}^{*-}=\text{Re}+\text{O}+\text{2}{\text{O}}^{\text{2}-}$$ (11)

**Fig 11:**
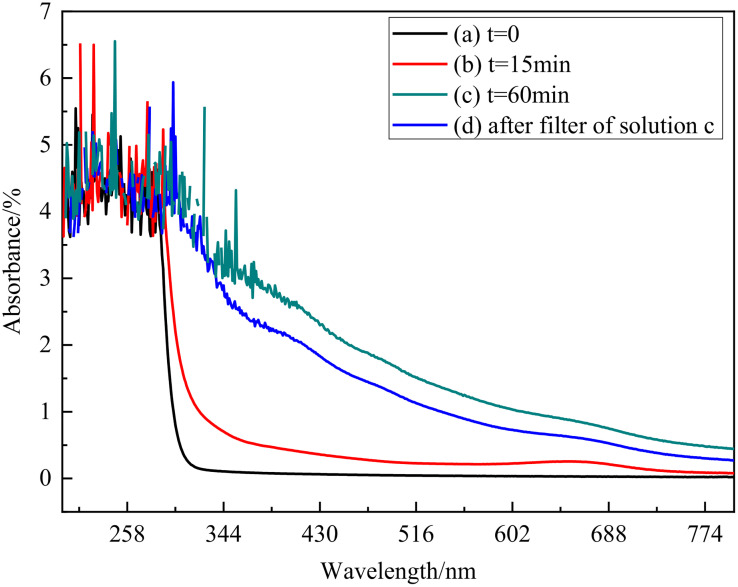
UV-vis absorption spectra of electrolyte with discharge time a t = 0, b t = 15 min, c t = 60 min, d filtered solution of solution c.

The standard electrode potentials used for electrochemical reactions (6)–(9) were taken from the reports in Rojas and measurement from Bard [42–44]. under standard conditions (25°C, 1 M concentration, and 1 atm pressure). The cathodic plasma supplies high-energy electrons that enable otherwise unfavorable reduction pathways beyond equilibrium limits. Therefore, from an electrochemical perspective, the cathodic potential is significantly more negative than the standard electrode potential of reactions (6) to (9), leading to the predominance of reaction (6). The occurrence of reaction (6) will result in the formation of ReO_3_, aligning with the experimental findings. The ReO_3_ present in the product primarily originates from reactions (4)–(6). In contrast, the formation of metallic Re is attributed to three potential pathways: (1) ReO_4_^-^ ions that migrate to the limited cathode discharge zone undergo reduction via reactions (10) and (11) with high-energy electrons; (2) direct reduction of ReO_4_^-^ via reaction (7); and (3) secondary reduction of ReO_3_ by H_2_ (generated from H⁺ reduction at the cathode).The reactions can be divided into three parts including energy input, electrode kinetics and thermodynamic coupling, chemical and electrochemical reactions. First, cathodic plasma dominates energy input, overriding electrochemical equilibrium and enabling ReO_4_^-^ reduction that is not feasible in traditional electrolysis [45]. Second, the high energy density at the cathode causes sputtering of the W electrode, introducing W^3+^ ions into the electrolyte (as observed in Fig 1); simultaneously, plasma-driven local heating synergizes with electrochemical reactions to accelerate ReO_3_ nucleation. Finally, a series of chemical and electrochemical reactions (Eqs. (1)–(11)) occur, leading to the formation of Re and ReO_3_.During the discharge process in solution of 0.1M NH_4_ReO_4_ − 0.2M H_2_SO_4_, the solution was collected under different discharge stage for analysis of UV-vis spectrum as shown in Fig 11.

A filtered-supernatant control was used in UV-vis measurements to eliminate the effect of scattering by fine particles. The spectra were normalized based on the path length, dilution factor, and sample mass to ensure accurate comparison between different experimental conditions. The emission spectra were background-subtracted using a baseline correction method. The spectra were then normalized to the maximum peak intensity to account for variations in plasma emission strength during the electrolysis process. The signal at 200 ~ 300 nm comes from NH_4_ReO_4_ [15], and it shows no response at wavelength greater than 300 nm. However, the solution with generated particles shows absorption from 300nm to 800nm, and the intensity is enhanced with discharge time. The result of uv-vis spectra indicates that the generated product exhibits broader absorption, significantly enhanced in the visible light range. Even though through filter, solution d still exhibits a strong absorption compared with solution a and solution b, which means the fine particles probably remain in the electrolyte. To account for scattering, the UV-vis spectra were treated using the Kubelka–Munk function to separate the absorption effects from scattering contributions. The data suggest that the observed absorption increase in the 300–800 nm range is partly due to scattering, with significant absorption from the ReO_3_ species occurring between 600–700 nm [46]. Moreover, it can be proved further that the product in our work is mainly composed of ReO_3_ by the absorption between 600nm and 700nm. By the measured optical data, UV-vis spectral, XRD and SEM results, ReO_3_ formation pathways of ReO_4_^-^ → ReO_3_+  → ReO_3_ is suggested, and composition and morphology adjustment can be guided through the optical data, electrical signal and SEM results. It can be found that lowering voltage suppresses W sputtering and promotes ReO_3_ dominance in Fig 4, moreover H_2_SO_4_ addition reduces particle aggregation by enhancing H-bonding coordination in Fig 5.

## Conclusions

This study successfully synthesized ultrafine metallic Re and ReO_3_ particles using a non-contact solution plasma electrolysis technique. In our experiments, plasma electrolysis provides a novel energy source in the form of high-energy electrons, which enable the reduction of normally non-spontaneous reactions such as ReO_4_^-^ → ReO_3_. These electrons are accelerated by the plasma field, providing the necessary energy to overcome electrochemical potential barriers and promote the reduction of ReO_4_^-^ to ReO_3_. The experimental results revealed that increasing the NH_4_ReO_4_ concentration enhanced the ReO_3_ content in the product. The addition of H_2_SO_4_ resulted in well-dispersed particles with reduced interparticle adhesion, yielding spherical particles with an average diameter of approximately 400 nm; this phenomenon was attributed to hydrogen-bonding coordination, which acts as a steric barrier to prevent particle aggregation. Freeze-drying of the electrolyzed products produced block-like structures with rod-shaped clusters grown on their surfaces, where the rods measured ~500 nm in length and ~100 nm in diameter. Voltage measurements demonstrated that the anode voltage remained within 1–2 V regardless of the applied potential, with the voltage drop concentrated in the cathode plasma region. The formation of ReO_3_ involved the following reactions:

1. ReO_3_^+^ + e^-^ → ReO_3_
2. H_2_OReO_3_^+^ + e^-^ → ReO_3_ + H_2_O
3. ReO_4_^-^ + 2H^+^ + e^-^ → ReO_3_ + H_2_O

The generation of metallic Re was governed by:

1. ReO_4_^-^ + 8H^+^ + 7e^-^ → Re + 4H_2_O
2. ReO_4_^-^ + e^*-^ → Re^+^ + 2O + O_2_^-^
3. ReO_4_^-^ + 3e^*-^ → Re^+^ + O + 2O_2_^-^

## Supporting information

S1 Fig Particle size distributions extracted from SEM images in Figs 1, 3 and 4. (DOCX)

S1 Data Raw data. (ZIP)
