# Supplementary material for: An exploratory study on the preparation of metallic rhenium (Re) and ReO3 via non-contact solution plasma electrolysis
Source: PLoS One. 2025 Dec 8;20(12):e0338178. doi: 10.1371/journal.pone.0338178 (PMC12685202; doi:10.1371/journal.pone.0338178)
Supplement: S1 Fig — (DOCX) [file pone.0338178.s001.docx]

S1 Fig. Particle size distributions extracted from SEM images
