# Supplementary figures and images for: An exploratory study on the preparation of metallic rhenium (Re) and ReO3 via non-contact solution plasma electrolysis
Source: PLoS One. 2025 Dec 8;20(12):e0338178. doi: 10.1371/journal.pone.0338178 (PMC12685202; doi:10.1371/journal.pone.0338178)

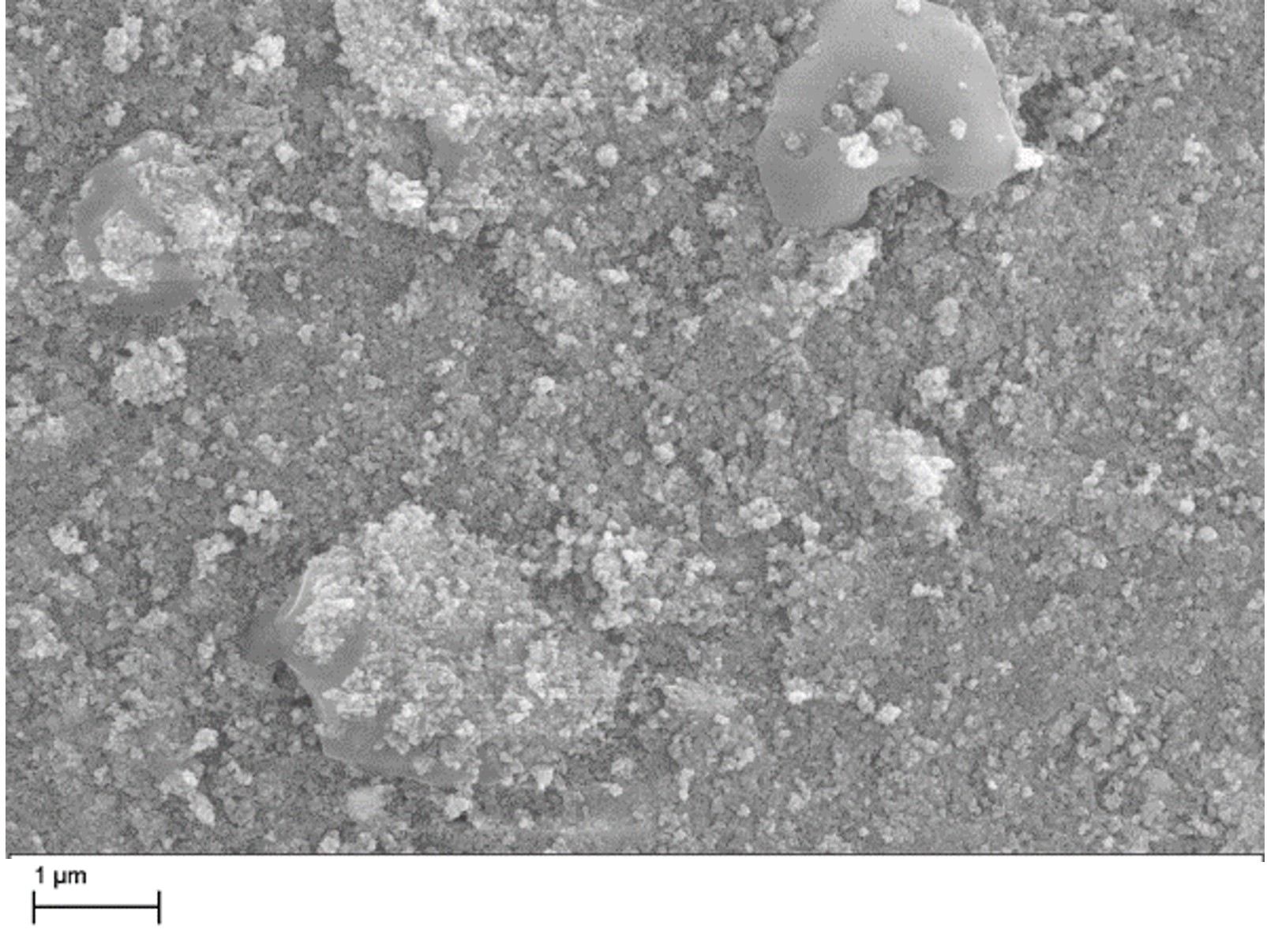

Supplement: S1 Data — (ZIP) [file pone.0338178.s002.zip › raw data/SEM/Fig.1 b.jpg]

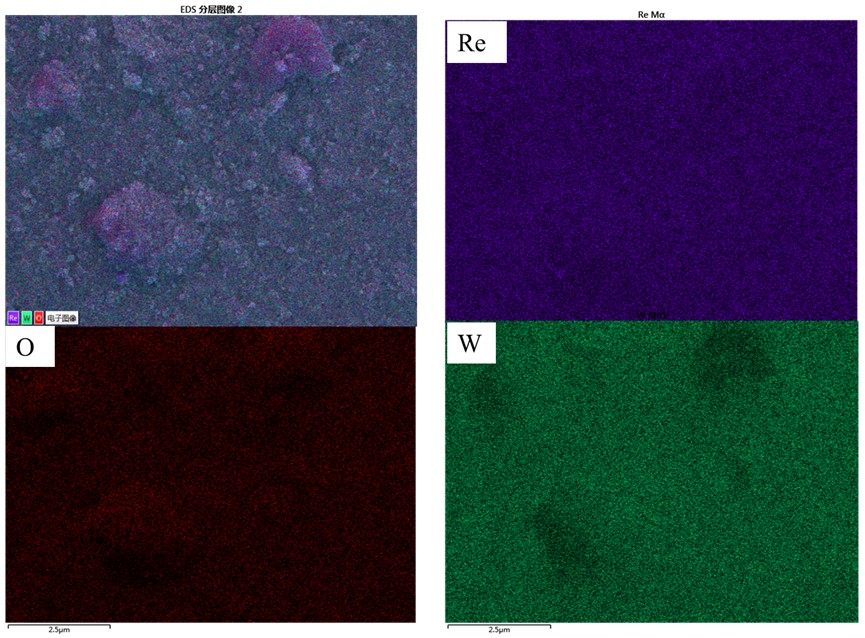

Supplement: S1 Data — (ZIP) [file pone.0338178.s002.zip › raw data/SEM/Fig.2.jpg]

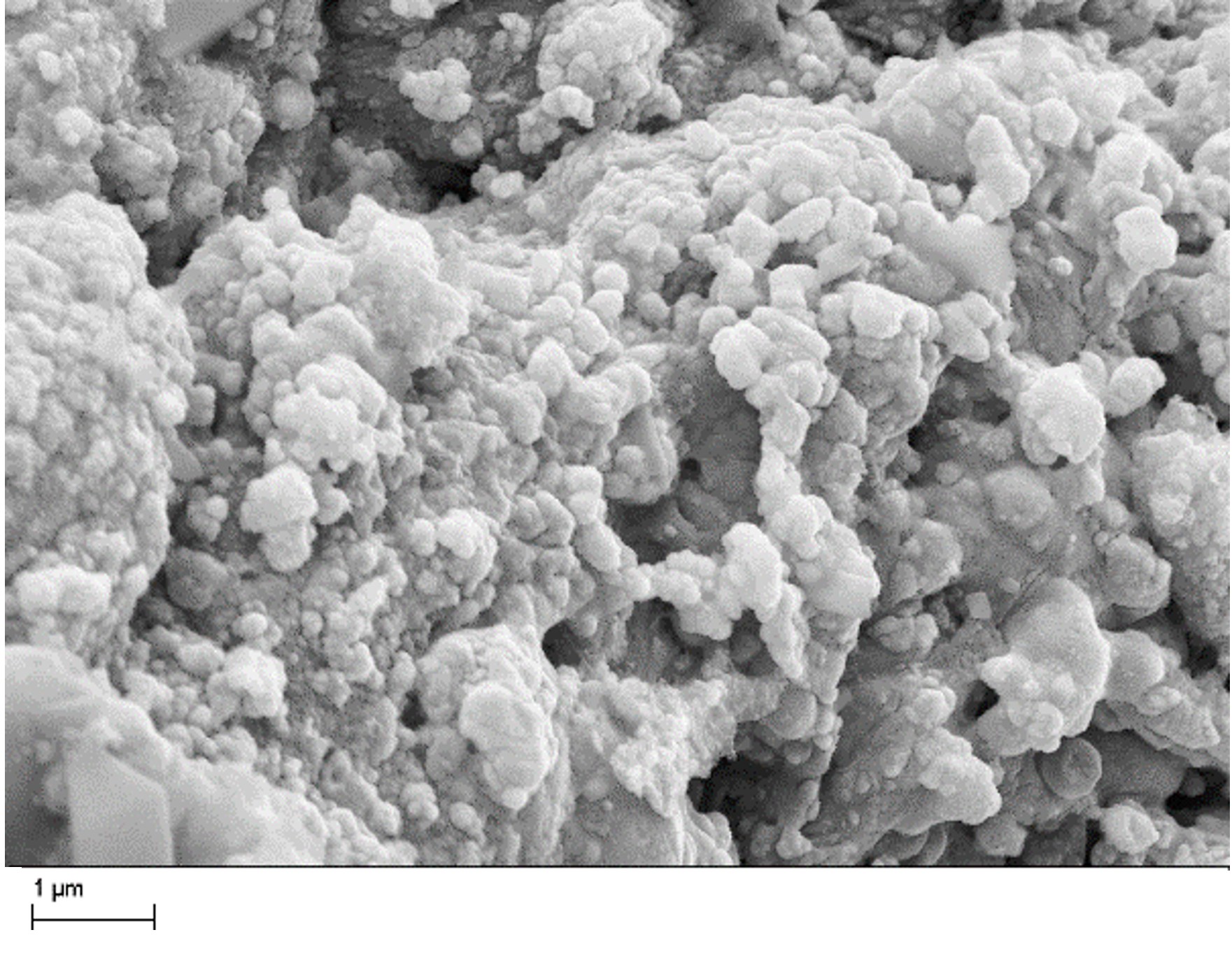

Supplement: S1 Data — (ZIP) [file pone.0338178.s002.zip › raw data/SEM/Fig.3b.jpg]

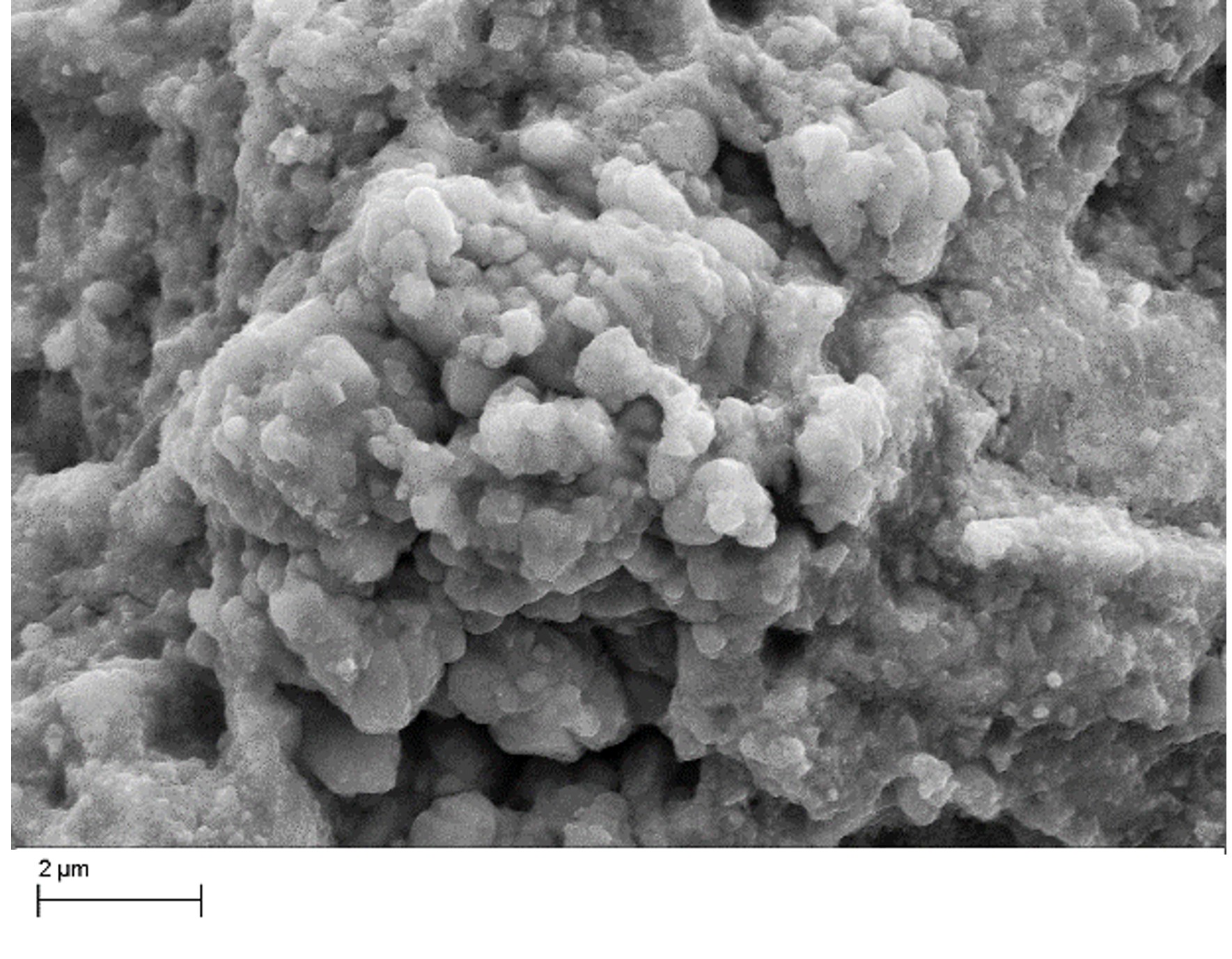

Supplement: S1 Data — (ZIP) [file pone.0338178.s002.zip › raw data/SEM/Fig.4a.jpg]

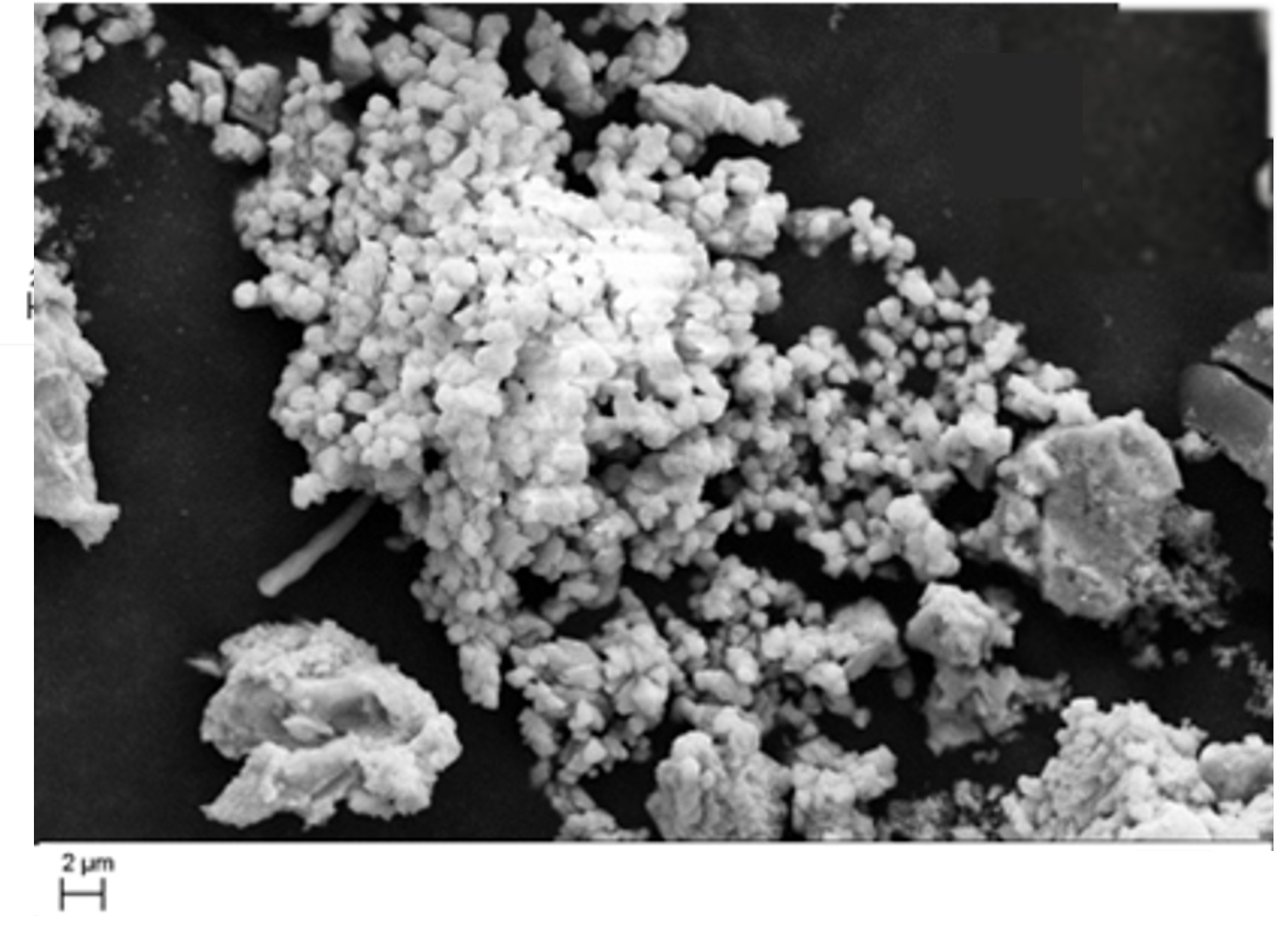

Supplement: S1 Data — (ZIP) [file pone.0338178.s002.zip › raw data/SEM/Fig.5a.jpg]

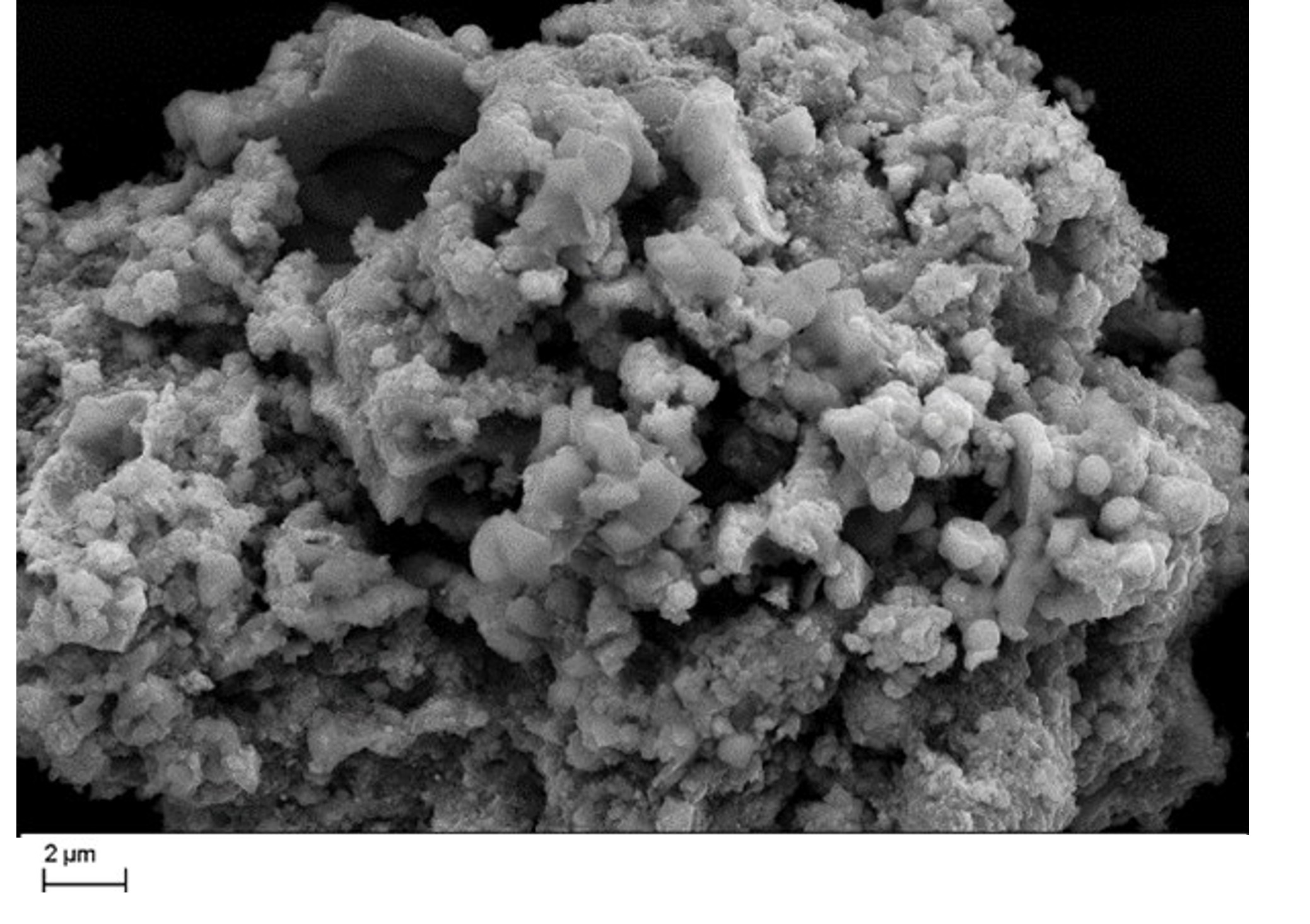

Supplement: S1 Data — (ZIP) [file pone.0338178.s002.zip › raw data/SEM/Fig.5b.jpg]

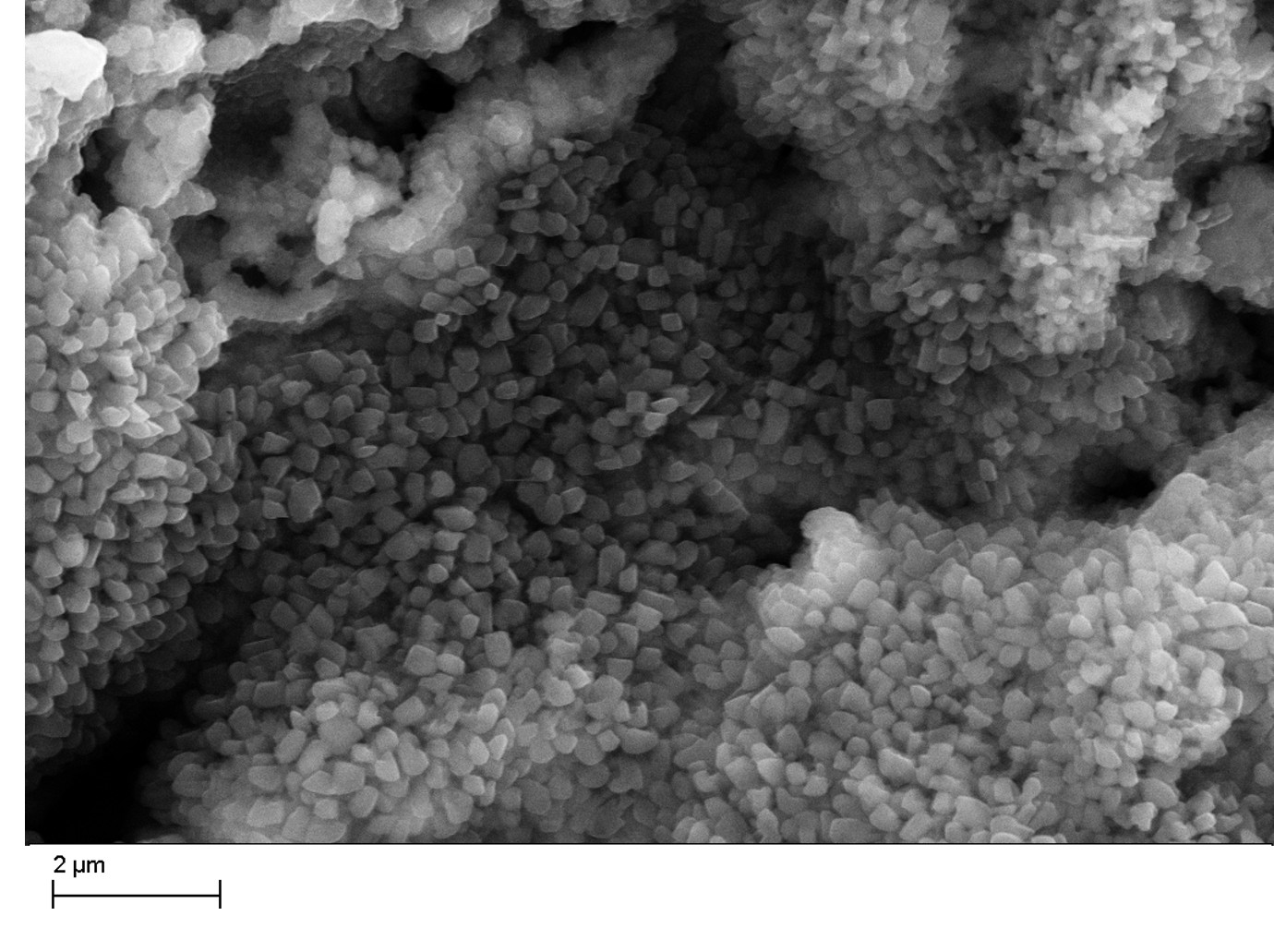

Supplement: S1 Data — (ZIP) [file pone.0338178.s002.zip › raw data/SEM/Fig.6b.jpg]

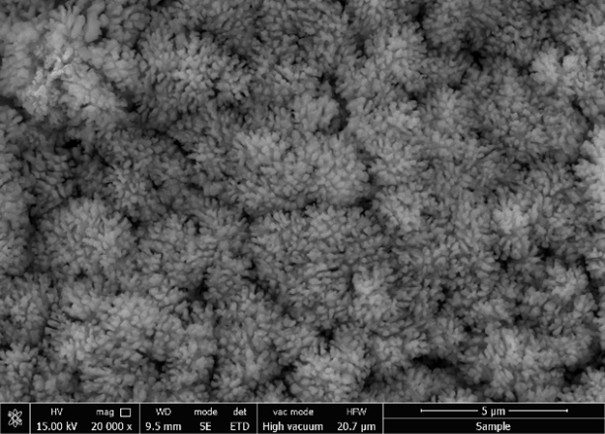

Supplement: S1 Data — (ZIP) [file pone.0338178.s002.zip › raw data/SEM/Fig.7a.jpg]

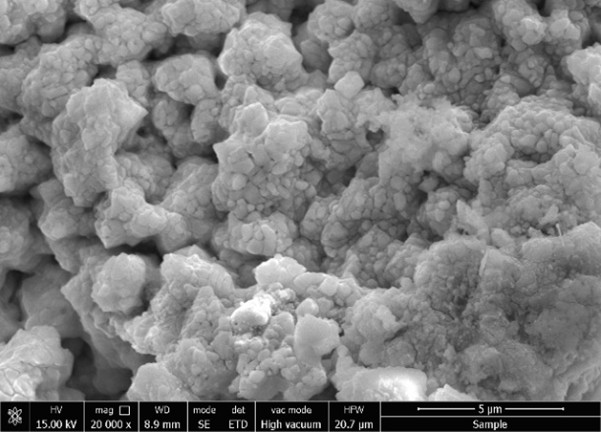

Supplement: S1 Data — (ZIP) [file pone.0338178.s002.zip › raw data/SEM/Fig.7b.jpg]
